# Foundations of circadian medicine

**DOI:** 10.1371/journal.pbio.3001567

**Published:** 2022-03-24

**Authors:** Achim Kramer, Tanja Lange, Claudia Spies, Anna-Marie Finger, Daniela Berg, Henrik Oster

**Affiliations:** 1 Charité –Universitätsmedizin Berlin, Laboratory of Chronobiology, Berlin, Germany; 2 University of Lübeck, Department of Rheumatology & Clinical Immunology, Center of Brain, Behavior and Metabolism, Lübeck, Germany; 3 Charité –Universitätsmedizin Berlin, Department of Anesthesiology and Intensive Care Medicine, Berlin, Germany; 4 Christian-Albrechts-University Kiel, Department of Neurology, Kiel, Germany; 5 University of Lübeck, Institute of Neurobiology, Center of Brain, Behavior and Metabolism, Lübeck, Germany

## Abstract

The circadian clock is an evolutionarily highly conserved endogenous timing program that structures physiology and behavior according to the time of day. Disruption of circadian rhythms is associated with many common pathologies. The emerging field of circadian medicine aims to exploit the mechanisms of circadian physiology and clock–disease interaction for clinical diagnosis, treatment, and prevention. In this Essay, we outline the principle approaches of circadian medicine, highlight the development of the field in selected areas, and point out open questions and challenges. Circadian medicine has unambiguous health benefits over standard care but is rarely utilized. It is time for clock biology to become an integrated part of translational research.

## Abstract 2

The emerging field of circadian medicine implements and translates findings from clock biology to improve health. Circadian medicine has clear health benefits over standard care but is rarely used owing to a lack of systematic and mechanistic evidence and overarching concepts. This Essay explains the principles of circadian medicine and highlights future approaches to promote its dissemination.

## Principles of circadian organization

**Fig 1:**
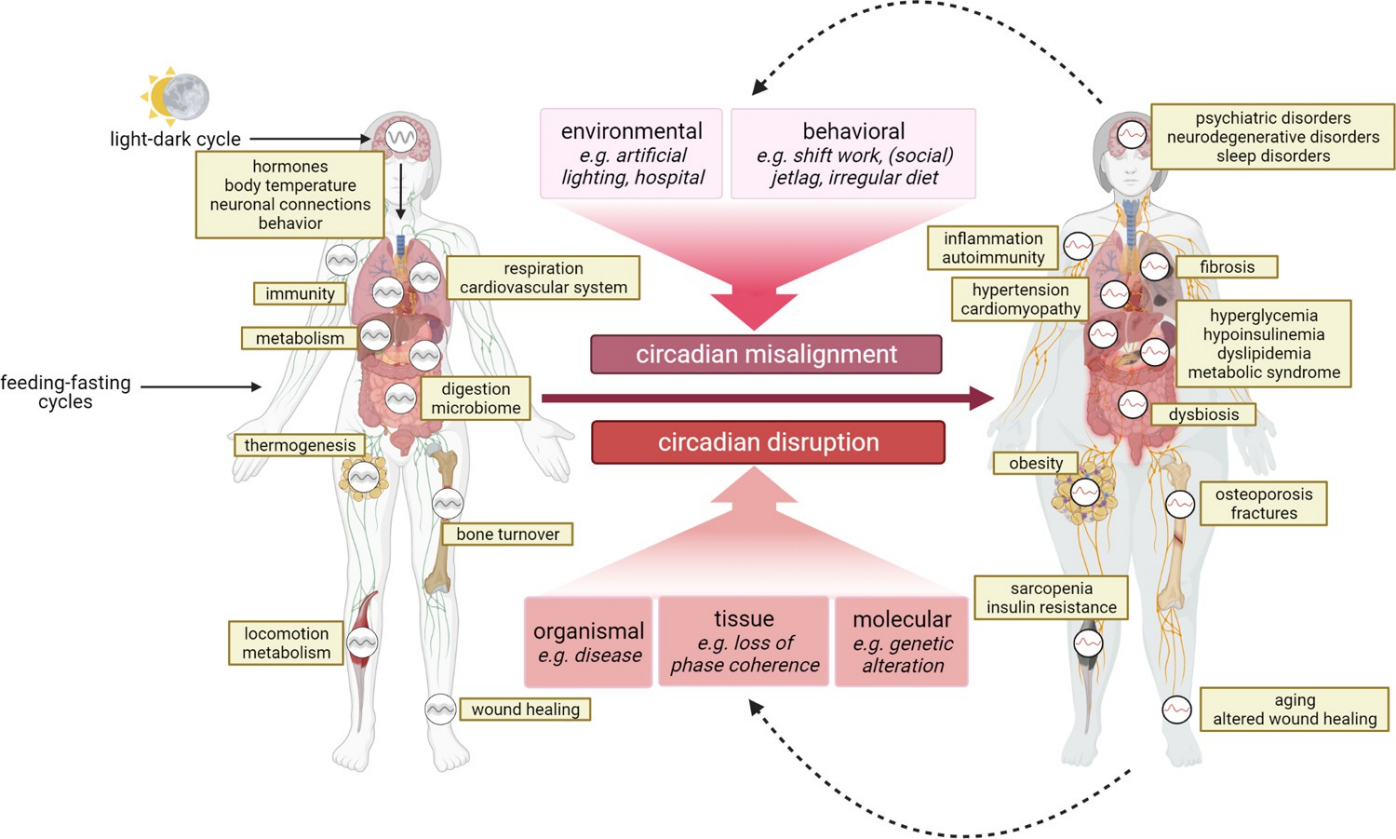
Circadian misalignment and disruption are associated with disease. Circadian rhythms are present at the level of the individual cell, tissue, and system and contribute to the optimal temporal coordination of physiological organ functions with environmental entrainment signals (left). It is therefore not surprising that misalignment of endogenous circadian and exogenous environmental cycles, as well as disruption of endogenous circadian rhythmicity is associated with or increases the risk for many diseases (right). Moreover, by contributing to circadian disruption or by altering behavioral rhythms, disease may in turn aggravate pathologies related to dysfunction of the circadian system (created with biorender.com).

Humans are a rhythmic species. During evolution, they have adapted to environmental cycles such as daily and annual rhythms to be able to anticipate recurring opportunities and challenges. Nearly all physiological processes such as immune responses and energy metabolism—but also many behavioral functions—are temporally modulated by endogenous timing systems. The best-studied biological timer is the so-called circadian clock (from the Latin *circa diem* meaning “about a day”), a fundamental biological program that is critical to health [1–3]. Circadian rhythm disorders are on the rise in our modern, 24/7 society and occur, for example, during shift work. They are associated with an increased risk of common diseases including psychiatric and neurodegenerative disorders, metabolic and cardiovascular disorders, immune system dysfunction, and some sorts of cancer (Fig 1). Before describing the current state of knowledge about the medical implications of the circadian clock, let us first consider some basic principles of circadian organization.

Circadian rhythms are ubiquitous and affect almost all biological functions. Prominent examples are sleep–wake and feeding–fasting cycles, but cognitive performance, glymphatic and lymphatic fluid flow, body temperature, insulin sensitivity, glucose metabolism, immune cell trafficking and activity, hormone secretion, blood pressure, heart rate, kidney function, adipose tissue physiology, and many more processes are also affected by circadian rhythms (for recent reviews, see [3,4]). The circadian clock governs molecular and cellular processes at virtually every level of regulation. We know from mouse experiments that between 5% and 20% of all protein-coding genes are rhythmically expressed in each tissue, and nearly half are rhythmic somewhere in the body [5]. This ubiquity of circadian rhythms is reflected at the protein [6] and metabolite levels [7]. Recent data reveal that even the composition of the gut microbiota exhibits prominent circadian rhythms [8]. The ultimate goals of such temporal structuring of physiology and behavior are the anticipation of predictable changes in environmental demands and the temporal segregation of physiologically incompatible processes.

Circadian clocks are cellular entities based on a set of core clock genes and proteins organized in interlocked transcriptional–translational feedback loops (TTFLs). In the core circadian TTFL, the transcription factors CLOCK (circadian locomotor output cycles kaput) and BMAL1 (brain and muscle ANRT-like 1) drive transcription of period (*Per1-3*) and cryptochrome (*Cry1/2*) genes during the day. PER and CRY proteins form complexes that negatively feedback on CLOCK/BMAL1 activity during the night, thus shutting down their own transcription. The core TTFL is stabilized by several accessory loops to create self-sustained oscillations that persist even under nonrhythmic environmental conditions. In other words, cycles in sleep and wakefulness, body temperature, and hormone levels are not driven by environmental rhythms such as light–dark cycles but are sustained (with an endogenous, genetically determined period) even in constant environmental conditions. Although this was already well established for rodent clocks at the beginning of the 20th century, it was not until the groundbreaking work of Jürgen Aschoff in the 1960s that the existence of an endogenous clock in humans was unequivocally demonstrated [9]. While being principally self-sustained, circadian clocks and rhythms are synchronized to environmental cycles. This process is called entrainment. It ensures that oscillatory processes stably align with environmental cycles, which means that they always occur at predictable times relative to environmental time cues. The dominant natural time signal (or zeitgeber) for the human circadian clock is light, but food signals can also entrain cellular circadian clocks in peripheral tissues [10]. Temperature rhythms, exercise, and hormones such as cortisol and melatonin can also be applied as zeitgebers, for example, to accelerate realignment of internal and external time after rapid travel across time zones, a process termed jetlag.

In the 1970s, Ron Konopka and Seymour Benzer identified the first clock gene mutants in the fruit fly *Drosophila*, thus establishing a genetic basis for behavior [11]. With the cloning of the fly period (*per*) gene in the 1980s, the molecular biology era of chronobiology began [12,13]. The components of the molecular clockwork were successively discovered in different species (in mammals, about a dozen genes are involved) and the molecular mechanism of circadian rhythm generation, the TTFL, was deciphered [14]. Over 25 years later, in 2017, these achievements were acknowledged with the Nobel Prize in Physiology or Medicine for Michael Rosbash, Jeff Hall, and Michael Young.

The human circadian system is hierarchically organized. A master clock resides in the suprachiasmatic nucleus (SCN) of the hypothalamus. It receives information on external light conditions from the eyes via the retinohypothalamic tract [4]. In the retina, light is perceived by rods, cones, and blue-light sensitive ganglion cells expressing the photopigment melanopsin to synchronize the SCN clock with the environment [15]. Through a variety of routes, the SCN synchronizes the many molecular clocks in other brain areas and in peripheral organs [3,4]. Almost every cell in our body harbors its own molecular clockwork. Coupling signals between the SCN and peripheral clocks, as well as between single-cell clocks within a tissue, provides coherence and stable phase relationships within the circadian system. It is believed that the pathological effects of circadian disruptors such as shift work, jetlag, and nocturnal light exposure are the consequence of internal desynchronization (the loss of phase coherence between different tissue clocks) [3].

The expression of circadian rhythms in humans shows large variations. Interactions between genetic disposition, age, sex hormones, and light exposure (intensity, duration, and timing) determines the so-called chronotype [16]. In early and late chronotypes, clock-controlled processes tend to occur early and late, respectively, in relation to the synchronizing environmental cycles. There is a striking age dependency of chronotype: Children on average start as early types but become later and later during puberty, whereas adults become earlier and earlier with increasing age [17]. Chronotype is approximately normally distributed within the population, indicating polygenic traits [18]. Several candidate genes have been identified based on linkage between polymorphisms and chronotypic variance. The best-known example is a variant in the clock gene period 2 (*PER2)*, which causes familial advanced sleep–wake phase syndrome (FASPS) [19].

The circadian system is tightly linked to sleep [20]. Sleep is promoted by a homeostatic process S, which controls sleep pressure as a function of wake time, and antagonized by process C, the circadian regulator of wakefulness. In humans, process C peaks in the afternoon and declines in the evening, opening the sleep window and allowing sleep to begin at a time of high sleep pressure. Sleep-regulating substances and wakefulness-inducing neurotransmitters act on subcortical sleep regulatory centers with strong connections to the SCN, but also to neurons of the stress system. Thus, the onset of sleep is associated with decreased activity of stress systems but is also characterized by darkness, recumbency, and decreases in food intake, locomotion, stimulus processing, and core body temperature. All these factors also act as timing signals for the SCN and peripheral clocks. In consequence, the manipulation of the circadian system always affects sleep and vice versa.

## The circadian clock and disease

Fundamental insights from chronobiology have enabled us to understand the relevance of the circadian system for health and disease. It is now widely accepted that a functional and well-aligned circadian clock promotes health, while circadian disruption increases disease risk (Fig 1). Four fundamental insights paved the road leading from chronobiology to circadian medicine.

### Disruption of the clock causes pathologies

In rodents, clock function has been experimentally manipulated at different levels and by various methods. The common result of such loss-of-function studies is that disrupting the clock leads to pathology. We refer to in-depth reviews and only list a few examples here. Genetic deletion of the essential clock gene *Bmal1* [14] in mice leads to various pathologies, including hyperglycemia, hyperlipidemia, increased inflammation, and premature aging [21]. *Bmal1* knockout in various tissues leads to phenotypes such as hypoglycemia and dyslipidemia (in the liver) [22], metabolic inefficiency and impaired triglyceride biosynthesis (in the muscle) [23], obesity (in adipose tissue) [24], or increased inflammation and insulin resistance (in myeloid cells) [25]. Variations of such phenotypes are seen after mutation or depletion of other clock genes. Anatomical ablation of the SCN pacemaker in rodents is associated with changes in intestinal immune cells and microbiota [26], increased adiposity [27], and tumor growth [28]. Exposing mice to a chronodisruptive environment such as repeated shifts in the light–dark cycle (simulating shift work or jetlag) increases mortality in aged animals [29], promotes tumor growth [30] and autoimmune processes [31], and dysregulates inflammatory responses [32].

### Epidemiological, genetic, and laboratory studies link the circadian clock to human disease

Circadian disruption due to artificial light, shift work, travels across time zones, and social jetlag (the misalignment of biological and social time) has been linked to a variety of human diseases, ranging from sleep disorders, psychiatric and cardiovascular diseases to systemic chronic inflammation, impaired immune responses against pathogens, higher tumor risk, and worsened reactions to allergens and autoantigens [3]. For example, the large epidemiological Nurses’ Health Study showed that cancer is associated with shift work and circadian rhythm disruption [33]. Shift work further increases the risk of dyslipidemia and hypertension, type 2 diabetes, heart attacks, and multiple sclerosis [34,35]. It disrupts natural rhythms of the gut microbiota, thus increasing the risk of inflammatory bowel disease [36]. Social jetlag is associated with increased alcohol and cigarette consumption [37] and a higher prevalence of obesity [37], diabetes, cardiovascular diseases, and cancer [38]. Genetic association studies indicate a role for clock genes in immune, metabolic, and psychiatric disorders. For example, variants in several clock genes (including *BMAL1* and *PER2*) as well as in the melatonin receptor 1B (*MTNR1B*) gene are associated with an increased risk of specific metabolic disorders. In laboratory studies in humans, circadian misalignment leads to increased blood pressure, increased ghrelin, and decreased leptin levels, as well as reduced insulin sensitivity in skeletal muscle (for a review, see [39]).

### Disease occurrence and symptoms vary with time of day

The circadian clock controls a variety of physiological processes. Thus, it is not surprising that the expression of disease symptoms often also varies with the time of day. Some prominent examples for such rhythms are symptoms in asthma, allergic rhinitis, cancer, arthritis, pain, and depression as well as the incidence of heart attacks, suicidal intent, and peptic ulcers [2]. Epidemiological studies show a steep increase in the incidence of heart attacks and strokes in the morning hours [40]. Patients with rheumatoid arthritis (RA) are particularly affected by joint stiffness and pain in the morning hours, an aftereffect of a nocturnal increase in proinflammatory cytokines [41]. In asthma and allergic rhinitis, inflammatory processes and their symptoms are highest during the second half of the night—likely due to combined direct effects of sleep loss and circadian rhythms on pulmonary and nasal epithelial functions [42]. In neurodegenerative diseases, there are rhythms in mood and emotional volatility. The “sundowning syndrome” in patients with Alzheimer’s disease is a prominent example [43]. These patients are increasingly agitated and emotionally unstable in the late afternoon or evening. On the other hand, the “dawn phenomenon” in patients with diabetes, characterized by abnormally high fasting blood glucose levels due to impaired insulin secretion, is another example for an aggravation of disease symptoms during the morning [44].

### Circadian rhythms are disrupted in many pathologies

An overwhelming body of evidence shows that daily rhythms are disrupted in a wide range of diseases. Again, we can only give a few examples here. In patients with neurodegenerative diseases, such as Parkinson’s disease, Alzheimer’s disease, or Huntington’s disease, sleep–wake behavior, melatonin secretion, and clock gene expression rhythms are all disrupted [45]. This holds true for schizophrenia spectrum and several other psychiatric disorders [46]. In patients with autoimmune diseases, daily rhythms in rest–activity, hormone, and cytokine levels, as well as molecular clock rhythms are disrupted [47]. In type 2 diabetes, circadian regulation of insulin secretion is impaired [48] and shifted eating patterns correlate with increased body mass index [49], in line with disrupted gut microbiota rhythms [8].

## The triad of circadian medicine

**Fig 2:**
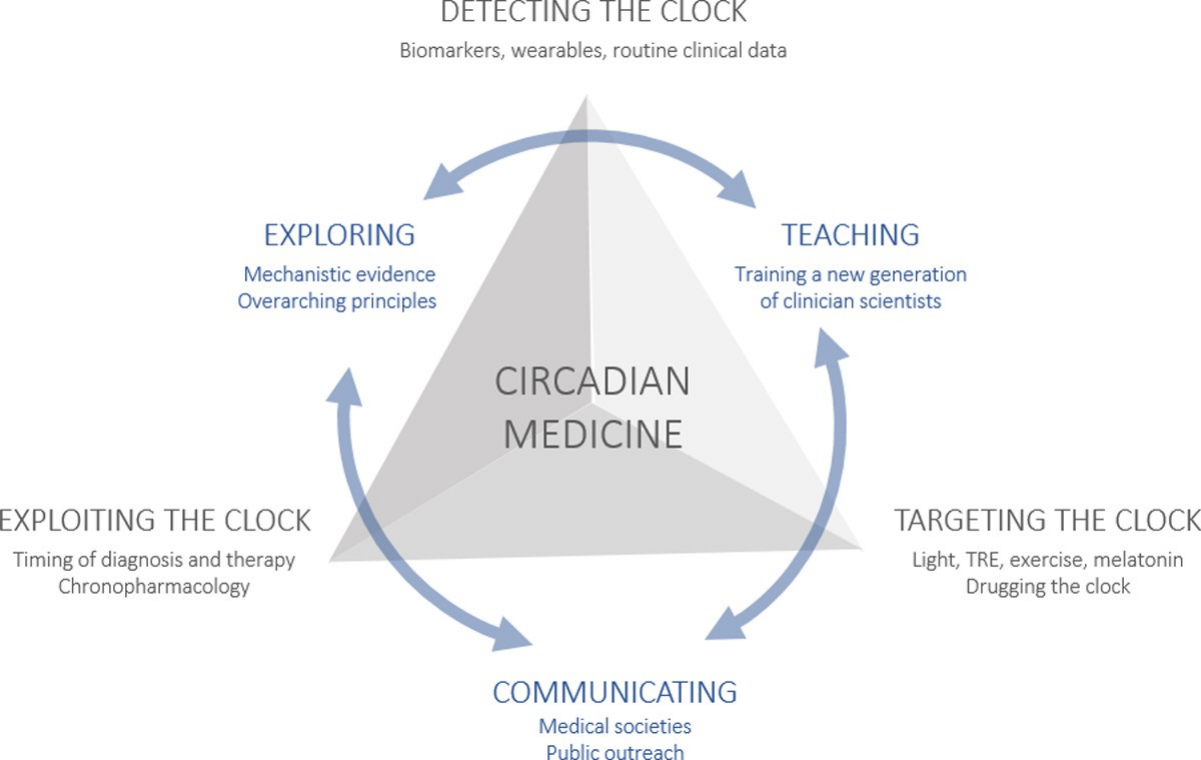
The future of circadian medicine. Our vision for the future of circadian medicine is to firmly integrate it into medical guidelines so that, for each patient, the circadian aspect is always taken into consideration and incorporated into the treatment plan as appropriate. Circadian medicine has many facets and to sustain its development, they all need to be advanced. They include treatment and diagnosis at the right time of day (exploiting), realigning and strengthening the circadian system (targeting), and diagnosing the patient’s circadian characteristics to develop personalized treatment plans (detecting). To achieve this goal, more evidence, especially on overarching principles of circadian medicine, needs to be gathered. To do this, on the one hand, we need a transdisciplinary approach to research, but we also need to train a new generation of physicians and communicate our findings more to the public and decision makers. TRE, time-restricted eating.

The findings described above have led to the development of circadian medicine. While first successes are becoming apparent, circadian knowledge is still far from being part of routine medical practice (Box 1). Circadian medicine essentially comprises 3 main approaches (Fig 2): using knowledge of physiological rhythms for time-of-day adapted treatment regimens (exploiting the clock); improvement or resynchronization of disrupted rhythms through interventions in the clock (targeting the clock); and development of circadian medicine as part of precision medicine through new diagnostic tools that allow personalized interventions tailored to the chronotype (detecting the clock).

**Box 1. A physician’s viewpoint on circadian medicine**

The lack of awareness of our internal clock and the processes it controls can easily be linked to the conveniences and challenges of modern societies. Opportunities and 24/7 offerings in cities that never sleep, and the associated work demands, have resulted in a lost sense of physical needs that are less urgent than, for example, the desire to grab a quick bite to eat at a temptingly advertised fast food joint in the middle of the night. Similarly, disciplined adherence to timely or chronomedical medication intake is usually achieved only when the effects of delayed intake are severe (for example, off-times in patients with Parkinson’s disease) or when the adverse effects of postponed nonchronomedical medication intake are severe (for example, insomnia after delayed intake of corticosteroids). Adherence to an individual’s circadian rhythm, along with a healthy, balanced diet, regular physical activity, and healthy sleep, has the inestimable value of having a lasting positive impact on health and healthy aging, but also the challenge of making personal lifestyle choices that can then be translated into personalized therapies as needed. To firmly implement circadian principles in medical practice will require compelling science, clear information that permeates all levels of society, and implementation of knowledge about circadian medicine in the training of medical professionals.

### Exploiting the clock

In most pharmacological studies in which the time of administration is explicitly evaluated for clinical outcome, there is a clear time-of-day dependent variation in drug efficacy or toxicity [2,50–53]. This applies to drugs targeting major common diseases such as hypertension, cancer, asthma, and arthritis. This variation is explained by daily rhythms in pharmacokinetics and pharmacodynamics of most short-lived drugs [2,50,54]. For example, more than 50 years ago, HMG-CoA reductase, a rate-limiting enzyme of cholesterol synthesis, was described as being rhythmically expressed in rat liver. Its inhibitor, simvastatin, was approved some 20 years later for the treatment of hyperlipidemia with an explicit recommendation for the time-of-day to take it (“in the evening”)—one of the first chronopharmacological approvals. Timed immunotherapy in patients with melanoma has proven superior to standard therapy in terms of adverse effects and survival [55]. In RA therapy, application of a modified-release prednisone in the evening (with drug release during the second half of the night) reduces peak nocturnal interleukin-6 levels, thus positively affecting clinical parameters [42]. Such pharmacological studies are still few, although numbers have increased in recent years. Likewise, many surgical interventions can benefit from chronobiological optimization. For example, major adverse cardiovascular events are lower when aortic valve replacement is performed in the afternoon compared with morning surgeries, probably because myocardial tolerance to ischemia-reperfusion is better at this time of day [56]. Circadian effects are also important for disease diagnosis. For example, for the diagnosis of Cushing’s syndrome, which is characterized by hypercortisolemia, cortisol levels should be determined in the evening when endogenous cortisol production is at its trough [57], while adrenal insufficiency should be diagnosed in the morning when cortisol normally peaks [58].

### Targeting the clock

**Fig 3:**
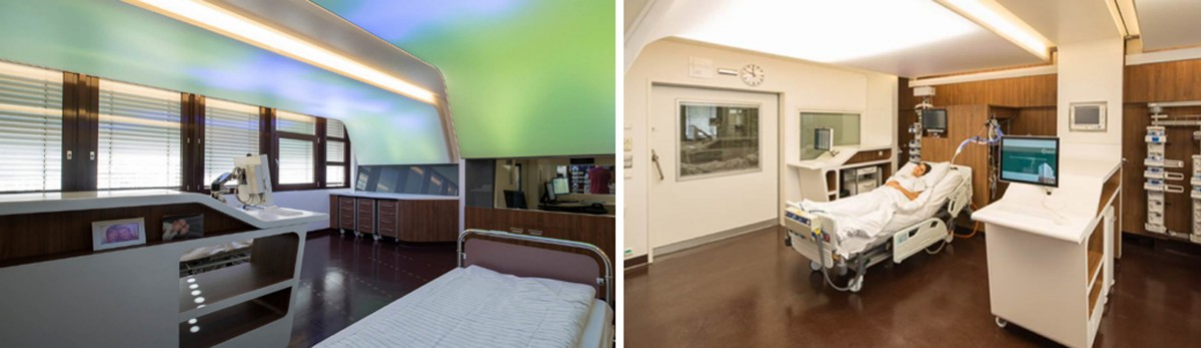
Circadian medicine in ICUs. Critically ill patients frequently exhibit disturbed or absent diurnal rhythms. This is likely to be a result of their illness and sedative measures but could also be related to the often-arrhythmic environment of an ICU (inadequate light exposure, parenteral nutrition, etc.). At the same time, they are arguably the best monitored patients, and vast amounts of routine clinical data are available with time courses for a variety of physiological parameters. We propose evaluating this treasure trove of information much more systematically with data science methods to detect the clock and improving light settings in the ICUs to target the clock. The analysis of clinical source data will help to characterize patients in the ICU in terms of their circadian rhythms, to identify predictors of circadian disruption as well as therapy-based improvements, and to associate circadian rhythms with clinical parameters (for example, delirium). In addition, since current ICUs do not provide adequate light settings, we have implemented a novel concept at Charité –Universitätsmedizin Berlin, consisting of a light ceiling for each experimental ICU bed that extends from the head above the patient to the patient’s feet [67]. Each light ceiling consists of multiple layers of light-emitting diodes designed to target the patient’s circadian clock. ICU, intensive care unit. *Photos can be used with kind permission of GRAFT; photo credit is given to Tobias Hein*.

Circadian rhythm disruption is not only a symptom, but also a risk factor for many diseases. In consequence, therapeutic approaches aimed at strengthening circadian rhythms may affect disease progression or even prevent disease initiation (Fig 3). Many zeitgebers known to influence the circadian system, such as light, food, melatonin, and exercise, have been used for this purpose [59]. Light therapy is a widely used adjunct therapy in sleep–wake rhythm and psychiatric disorders (such as depression and bipolar disorder [60]), but also in neurodegenerative disorders (such as Alzheimer’s disease and Parkinson’s disease [61]). Time-restricted eating, in which food consumption is limited to a window of 8 to 12 hours per day (without caloric restriction), improves glucose regulation, triglyceride levels, blood pressure, and various quality of life measures [62]. Exercise can phase-shift the human clock in a similar manner to light and enhance circadian amplitude of rest–activity cycles, body temperature, and muscle strength [63]. Furthermore, the circadian system can also be addressed by pharmacological means (“drugging the clock”), not only to strengthen clock rhythms, but also to address specific links between the clock and disease. Recently, several small molecule modulators of core clock function have been developed. They alter clock period, enhance its amplitude, or affect a specific clock component. For example, molecules that stabilize the clock protein cryptochrome 1 (CRY1) improve glucose tolerance in obese mice [64,65], while inhibiting casein kinase 1δ (CK1δ) prolongs clock period and may be used to treat (F)ASPS [66]. Of note, however, none of these molecules has yet been shown to be effective in humans.

### Detecting the clock

In humans, the interaction of genetic disposition, age, sex, and light exposure (intensity, duration, and timing) determines the entrained phase, the so-called chronotype [17]. Since the outcome of many therapeutic interventions depends on internal circadian phase, such interventions should be tailored to the individual chronotype. The 2 main prerequisites for successful application of circadian precision medicine are attaining a high degree of accuracy, sensitivity, and reliability of individual circadian phase assessment in clinical practice and a reasonable cost and practical applicability. Chronotype is traditionally determined by questionnaires, actigraphy, or by determining the timing of the evening rise in melatonin levels in blood or saliva [68]. Recently, more practicable biomarkers have been suggested that use single blood or tissue samples instead of time series analyses, which may allow the determination of chronotype in larger cohorts [69]. In addition, we anticipate that wearables and telemedicine will increasingly contribute to circadian diagnostics, prevention, and health maintenance. Future challenges include the use of clinical source data to characterize patients in hospitals with regard to their circadian rhythms (Fig 3), as well as the development of new diagnostic tools for circadian disruption. For example, shift work probably leads to internal desynchronization of tissue clocks, which has been very difficult to measure in animal models and virtually impossible in humans.

## Conclusion and open questions

In summary, overwhelming evidence indicates that the circadian clock is essential for health while its disruption causes pathologies [1]. These findings have sparked the development of circadian medicine, and the first successes in this field are becoming apparent. At the same time, in many disease areas, the extent, nature, and mechanism of circadian disruption and the mode of action of chronotherapies have not yet been sufficiently studied and validated. There is a lack of knowledge about the overarching principles of circadian disruption, its diagnosis, and therapy. To unravel these principles, several fundamental questions remain to be answered.

### How can circadian clock function and rhythms be measured in clinical settings?

Traditionally, daily rhythms are captured by measuring many data points over one or more 24-hour cycles. In some clinical settings, this is possible (for example, in intensive care units), but for broader application, simpler solutions such as wearables are needed. New biomarkers can already infer circadian phase (chronotype) from a single biosample, but such tests are not yet available for detection of circadian changes in patients. Genetic markers may additionally be used to infer rhythm alterations in certain clinical settings.

### How can circadian disruption be defined and quantified?

Many stressors in our modern society can affect circadian rhythms and “circadian disruption” is an umbrella term for very different modes of circadian alterations. Conceptually, one could distinguish at least 3 possible scenarios that are not mutually exclusive. The first is loss of rhythms or reduced rhythm amplitudes. The second is internal desynchronization, for example, different tissue clocks running with altered phase relationships. This is predicted when zeitgebers (such as light and food) are inconsistent (as may be the case with shift work). The third is circadian misalignment, for example, when internal synchrony is maintained but social demands do not match with internal time (such as when school starts too early for adolescents with late chronotypes). A common language is needed, and better tools to qualitatively and quantitatively describe circadian disruption under different conditions.

### How does circadian disruption affect disease risk?

How exactly do shift work or other circadian disruptions contribute to a higher risk of diseases? What are the molecular and cellular mediators? How does clock disruption interact with other factors such as sleep loss, a lack of light exposure, increased stress, or unhealthy diets? Experimental protocols that aim to separate the effects of the circadian system from those of other factors are extremely challenging. We predict that in most cases where circadian medicine leads to positive health outcomes, circadian rhythm modulations exert a joint, synergistic influence on behavioral and physiological parameters together with other effectors such as caloric restriction or improved sleep.

### How to choose and use the right circadian medicine approach?

To date, there are almost no recommendations or evidence-based guidelines on which type of circadian medicine should be used for which pathology, because evidence on the mechanisms of action is still sparse and no randomized controlled trials have been conducted.

### How can chronobiology inform personalized medicine?

People are genetically different with regard to their circadian clocks and downstream physiology. However, the chronotype of patients has so far not had a role in the design of treatment. With simpler diagnostic methods for circadian characterization, this could change, and a truly individualized precision circadian medicine could be established.

In summary, it is becoming increasingly clear that circadian medicine has unambiguous health benefits over standard care. However, it is still far from being part of routine medical practice [70], and to truly advance circadian medicine, coordinated action is needed at different levels (Fig 2). In particular, there is still a lack of systematic and mechanistic evidence and overarching transdisciplinary chronomedical concepts. It is time to fill these gaps in order to make circadian aspects an integral part of translational research and clinical practice.

## Abbreviations

BMAL1: brain and muscle ANRT-like 1
CK1δ: casein kinase 1δ
CLOCK: circadian locomotor output cycles kaput
CRY1: cryptochrome 1
FASPS: familial advanced sleep–wake phase syndrome
MTNR1B: melatonin receptor 1B
RA: rheumatoid arthritis
SCN: suprachiasmatic nucleus
TTFL: transcriptional–translational feedback loop
